# Growth performance and gut health of *Escherichia coli*-challenged weaned pigs fed diets supplemented with a *Bacillus subtilis* direct-fed microbial

**DOI:** 10.1093/tas/txaa172

**Published:** 2020-09-12

**Authors:** Sangwoo Park, Jung Wook Lee, Kevin Jerez Bogota, David Francis, Jolie Caroline González-Vega, John K Htoo, Tofuko Awori Woyengo

**Affiliations:** 1 Department of Animal Science, South Dakota State University, Brookings, SD; 2 Division of Animal and Dairy Science, Chungnam National University, Daejeon, Republic of Korea; 3 Department of Animal and Food Sciences, University of Kentucky, Lexington; 4 Department of Veterinary and Biomedical Sciences, South Dakota State University, Brookings, SD; 5 Evonik Operations GmbH, Hanau-Wolfgang, Germany; 6 Department of Animal Science, Aarhus University, Blichers Allé, Tjele, Denmark

**Keywords:** direct-fed microbial, growth performance, gut health, weaned pig

## Abstract

This study was conducted to investigate the effects of a direct-fed microbial (DFM) product (*Bacillus subtilis* strain DSM 32540) in weaned pigs challenged with K88 strain of *Escherichia coli* on growth performance and indicators of gut health. A total of 21 weaned pigs [initial body weight (BW) = 8.19 kg] were housed individually in pens and fed three diets (seven replicates per diet) for 21 d in a completely randomized design. The three diets were a corn-soybean meal-based basal diet without feed additives, a basal diet with 0.25% antibiotics (neo-Oxy 10-10; neomycin + oxytetracycline), or a basal diet with 0.05% DFM. All pigs were orally challenged with a subclinical dose (6.7 × 10^8^ CFU/mL) of K88 strain of *E. coli* on day 3 of the study (3 d after weaning). Feed intake and BW data were collected on days 0, 3, 7, 14, and 21. Fecal scores were recorded daily. On day 21, pigs were sacrificed to determine various indicators of gut health. Supplementation of the basal diet with antibiotics or DFM did not affect the overall (days 0–21) growth performance of pigs. However, antibiotics or DFM supplementation increased (*P* = 0.010) gain:feed (G:F) of pigs during the post-*E. coli* challenge period (days 3–21) by 23% and 24%, respectively. The G:F for the DFM-supplemented diet did not differ from that for the antibiotics-supplemented diet. The frequency of diarrhea for pigs fed a diet with antibiotics or DFM tended to be lower (*P* = 0.071) than that of pigs fed the basal diet. The jejunal villous height (VH) and the VH to crypt depth ratio (VH:CD) were increased (*P* < 0.001) by 33% and 35%, respectively, due to the inclusion of antibiotics in the basal diet and by 43% and 41%, respectively due to the inclusion of DFM in the basal diet. The VH and VH:CD for the DFM-supplemented diet were greater (*P* < 0.05) than those for the antibiotics-supplemented diet. Ileal VH was increased (*P* < 0.05) by 46% due to the inclusion of DFM in the basal diet. The empty weight of small intestine, cecum, or colon relative to live BW was unaffected by dietary antibiotics or DFM supplementation. In conclusion, the addition of DFM to the basal diet improved the feed efficiency of *E. coli*-challenged weaned pigs to a value similar to that of the antibiotics-supplemented diet and increased jejunal VH and VH:CD ratio to values greater than those for the antibiotics-supplemented diet. Thus, under *E. coli* challenge, the test DFM product may replace the use of antibiotics as a growth promoter in diets for weaned pigs to improve feed efficiency and gut integrity.

## INTRODUCTION

Postweaning diarrhea caused by *Escherichia coli* strains lead to tremendous economic losses in the swine industry due to decreased growth rate and increased mortality and morbidity of weaned pigs (Fairbrother et al., 2005; Pan et al., 2017). Enterotoxigenic *E. coli* (ETEC) K88+ is one of the major strains of *E. coli* that causes diarrhea in weaned pigs (Marquardt et al., 1999; Yang et al., 2014). Antibiotics have been widely used as a strategy for dealing with postweaning diarrhea (Fairbrother et al., 2005; Wang et al., 2016). However, due to public concerns on the potential risks to human health coupled with the development of antibiotic resistance (Kemper, 2008; Li et al., 2015; Park et al., 2016), there is a need to adopt antibiotic-free feeding systems for pigs for pork production. The adoption of antibiotic-free feeding systems for pigs requires the development of feed additives that can be used as alternatives to in-feed antibiotics (Pettigrew, 2006; Stein and Kil, 2006). One of the promising key members among these alternatives is direct-fed microbials (DFM), also known as probiotics (FAO/WHO, 2002). For a DFM to be beneficial, it should have at least one of the following functions in the gastrointestinal tract (GIT): 1) enhance the growth of beneficial bacteria, 2) prevent the colonization of GIT with pathogenic microorganisms, 3) increase the digestive capacity and lower the GIT pH, 4) improve mucosal immunity, or 5) enhance gut tissue maturation and integrity (de Lange et al., 2010). The DFM used in the swine industry are classified into three main categories, including lactic acid-producing bacteria, *Bacillus* species, and yeast (Kenny et al., 2011; NRC, 2012). Of these, *Bacillus* species form spores, which enable them to be thermostable and survive at low pH. *Bacillus* species also produce antimicrobial peptides that kill pathogenic microorganisms, modify the composition of GIT microorganisms that result in reduced competition for nutrients between host and microorganisms, and increase the production of mucin in GIT, which protect the GIT from invasion by pathogens and other toxins (Grant et al., 2018). Additionally, *Bacillus* species produce fiber-degrading enzymes that enhance the nutrient digestibility of plant feedstuffs-based diets (Liu et al., 2018).

A DFM product (*Bacillus subtilis* strain DSM 32540) that can potentially inhibit the growth of the main commercially relevant pathogens of swine, has a very high proliferation rate in the presence of bile, and can effectively digest cellulose has been recently developed (protected in International Patent Application WO 2019/002471). However, information is lacking on the effect of including this newly developed DFM in diets for weaned pigs on growth performance and gut health. The objective of this study was to evaluate the effects of including *B. subtilis* strain DSM 32540 in diets for ETEC K88-challenged weaned pigs on growth performance and indicators of gut health.

## MATERIALS AND METHODS

Experimental procedures were reviewed and approved by the Institutional Animal Care and Use Committee at South Dakota State University (# 17-051A).

### Experimental Diets

In this study, three experimental diets were fed to the pigs (Table 1). The three diets were: a basal diet without any feed additives, a basal diet with antibiotics (neomycin and oxytetracycline), or the DFM product (*B. subtilis;* DSM 32540; GutPlus; Evonik Nutrition & Care GmbH, Hanau-Wolfgang, Germany). The diets were isoenergetic and similar in nutrient content and formulated to meet or exceed the NRC (2012) recommended energy and nutrient requirements for nursery pigs. The diets were fed in mash form.

**Table 1.** Composition of the experimental diets (as-fed basis)

	Diet^*a*^		
Item	NC	PC	DFM
Ingredient, %			
Corn	48.70	48.70	48.70
Soybean meal, 46% crude protein	32.05	32.05	32.05
Whey powder	10.00	10.00	10.00
Soybean oil	2.84	2.84	2.84
Wheat bran	3.00	3.00	3.00
Limestone	0.41	0.41	0.41
Dicalcium phosphate	1.41	1.41	1.41
Salt	0.19	0.19	0.19
Vitamin premix^*b*^	0.05	0.05	0.05
Mineral premix^*c*^	0.15	0.15	0.15
L-Lysine·HCl	0.50	0.50	0.50
L-Threonine	0.20	0.20	0.20
DL-Methionine	0.30	0.30	0.30
L-Tryptophan	0.09	0.09	0.09
L-Valine	0.11	0.11	0.11
Neomycin + oxytetracycline	–	0.25	
DFM	–	–	0.05
Calculated nutrient composition			
Crude protein, %	20.49	20.49	20.49
Ether extract, %	5.35	5.35	5.35
Acid detergent fiber, %	3.39	3.39	3.39
Neutral detergent fiber, %	8.69	8.69	8.69
Electrolyte balance, mEq/kg	200	200	200
Net energy, kcal/kg	2,462	2,462	2,462
SID amino acid, %			
Lys	1.35	1.35	1.35
Met	0.55	0.55	0.55
Met + Cys	0.81	0.81	0.81
Thr	0.85	0.85	0.85
Trp	0.30	0.30	0.30
Ile	0.75	0.75	0.75
Val	0.92	0.92	0.92
Leu	1.48	1.48	1.48
His	0.45	0.45	0.45
Phe	0.84	0.84	0.84
Total Ca, %	0.75	0.75	0.75
Total P, %	0.68	0.68	0.68
Digestible P, %	0.35	0.35	0.35

SID, standardized ileal digestible.

^*a*^NC = negative control diet, PC = NC supplemented with 0.25% antibiotics, DFM = NC supplemented with 0.05% DFM product (*B. subtilis;* DSM 32540). Antibiotics and DFM were added on top of basal diet.

^*b*^Provided the following per kilogram of diet: 11,011 IU vitamin A, 1,652 IU vitamin D_3_, 55 IU vitamin E, 0.04 mg vitamin B_12_, 4.4 mg menadione, 9.9 mg riboflavin, 61 mg pantothenic acid, 55 mg niacin, 1.1 mg folic acid, 3.3 mg pyridoxine, 3.3 mg thiamin, and 0.2 mg biotin.

^*c*^Provided the following per kilogram of diet: 165 mg Zn as ZnSO_4_, 23 mg Fe as FeSO_4_; 17 mg Cu as CuSO_4_, and 44 mg Mn as MnSO_4_.

### Experimental Animals and Procedure

A total of 21 pigs (Large White-Landrace female × Duroc male from Pig Improvement Company) weaned around 21 d of age and with an initial body weight (BW) of 8.19 ± 0.77 kg were obtained from a commercial farm and housed individually in 21 pens in the rooms of the Animal Resource Wing, South Dakota State University. The pigs were fed three experimental diets for 21 d in a completely randomized design (seven pens per diet).

All pigs were orally challenged with freshly grown K88 strain of *E. coli* on day 3 of the study as described by Lin et al. (2013). Diets and freshwater were offered to pigs ad libitum during the entire period. Pigs were observed four times per day during the experimental period for signs of illness, including diarrhea, lethargy, and dehydration. Animal BW and feed intake were determined on days 0, 3, 7, 14, and 21 of the study to calculate average daily gain (ADG), average daily feed intake (ADFI), and gain:feed (G:F). The occurrence and severity of postweaning diarrhea were assessed daily throughout the study on a pen basis by using the following fecal scoring system: 1 = firm feces, 2 = soft feces, 3 = mild pasty diarrhea, 4 = pasty diarrhea, 5 = watery diarrhea and dehydration, and 6 = most severe condition.

At the end of the feeding trial, all pigs were euthanized, and the following procedures took place. The gastrointestinal tract was divided into stomach, small intestine, cecum, and colon by using clamps to minimize digesta movement. The small intestine was stripped free of its mesentery and further divided into three sections: 1) duodenum (from pylorus to 80 cm distal to the pylorus), 2) ileum (from the ileal–cecal junction to 80 cm cranial to this junction), and 3) jejunum (the rest of the small intestine). A segment of 2 cm was collected from the middle of jejunum, and from ileum at 15 cm proximal to the ileo–cecal junction. The collected segments were prepared as described by Woyengo et al. (2011) for the determination of gut histomorphology. Samples of digesta from the distal ileum and the cecum were collected from each pig aseptically into sterile plastic containers. The ileal and cecal digesta samples were used to determine ileal and cecal pH; cecal digesta samples were then stored frozen at −20 °C for later determination of volatile fatty acid (VFA) concentration. The stomach, all sections of the small intestine, cecum, and colon were emptied of their digesta and weighed. Also, the spleen and liver were obtained, blotted dry with paper towels, and weighed.

### Sample Analyses

Crude protein (method 990.0;AOAC International, 2007) and total amino acid analyses of diets were determined by ion-exchange chromatography with postcolumn derivatization with ninhydrin. Amino acids were oxidized with performic acid, which was neutralized with Na metabisulfite (Llames and Fontaine, 1994; Commission Directive, 1998). Amino acids were liberated from the protein by hydrolysis with 6 N HCl for 24 h at 110 °C and quantified with the internal standard by measuring the absorption of reaction products with ninhydrin at 570 nm. Tryptophan was determined by HPLC with fluorescence detection (extinction 280 nm, emission 356 nm) after alkaline hydrolysis with barium hydroxide octahydrate for 20 h at 110 °C (Commission Directive, 2000). Spore count in diets was determined by VDLUFA method 28.2.2 (VDLUFA, 1976).

Samples for histomorphology were analyzed for villous height (VH) and crypt depth (CD) as described by Woyengo et al. (2011). The villous height to CD ratio (VH:CD) was calculated. Cecal digesta samples for VFA analysis were thawed and centrifuged and the resulting digesta fluid was prepared and analyzed for VFA (acetate, propionate, butyrate, and branched-chain VFA) as described by Woyengo et al. (2016). The pH in the ileum and cecum digesta were determined using a pH meter (AB 15; Fisher Scientific, Pittsburgh, PA).

### Statistical Analysis

All data obtained from this study were subjected to analysis of variance using the GLM procedure of SAS (SAS Inst. Inc., Cary, NC). The initial BW was treated as a covariate. Means were separated by the Tukey test. The residual versus the predicted plot procedure in SAS was used to identify outliers. The frequency of diarrhea was analyzed using the FREQ procedure of SAS and treatments were separated using the Xi^2^ statistic. Significance and tendencies were set at *P* ≤ 0.05 and 0.05 < *P* ≤ 0.10, respectively, for all statistical tests.

## RESULTS

**Figure 1.**
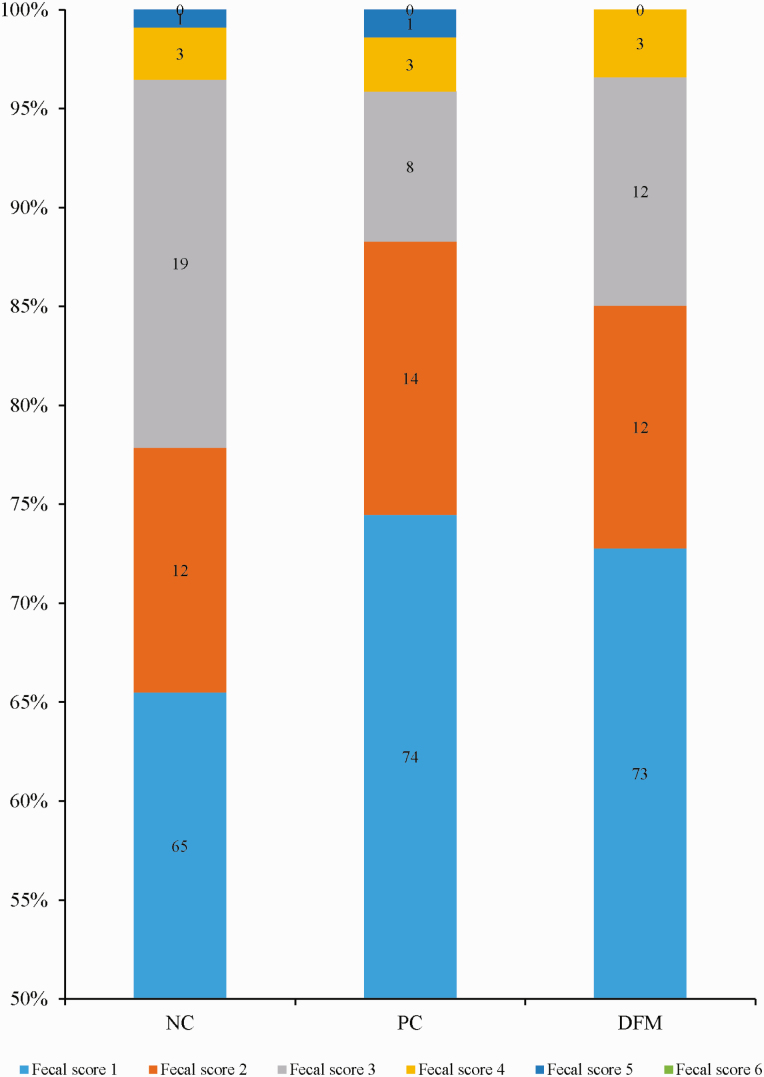
Proportion of fecal scores of pigs fed diets without or with antibiotics or DFM. Fecal score: 1 = firm feces, 2 = soft feces, 3 = mild pasty diarrhea, 4 = pasty diarrhea, 5 = watery diarrhea and dehydration, 6 = most severe condition. NC = negative control diet, PC = NC supplemented with 0.25% of antibiotics, and DFM = NC supplemented with 0.05% DFM product (*B. subtilis*; DSM 32540).

**Figure 2.**
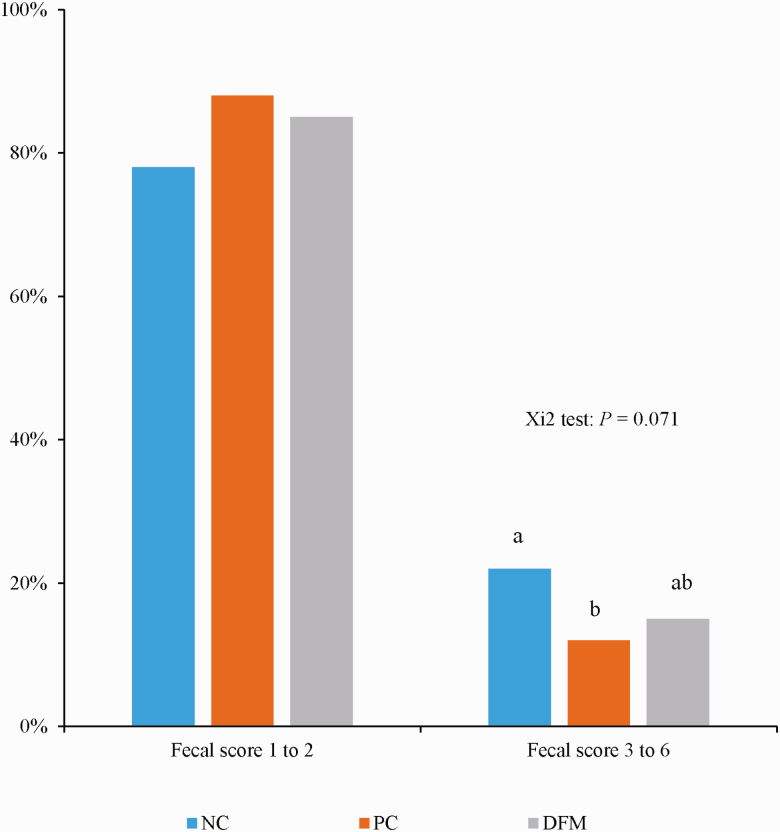
Frequency of diarrhea of pigs fed diets without or with antibiotics or DFM. Fecal score: 1 = firm feces, 2 = soft feces, 3 = mild pasty diarrhea, 4 = pasty diarrhea, 5 = watery diarrhea and dehydration, 6 = most severe condition. NC = negative control diet, PC = NC supplemented with 0.25% of antibiotics, and DFM = NC supplemented with 0.05% DFM product (*B. subtilis*; DSM 32540).

The analyzed crude protein values for the diets in Table 2 are close to the calculated values in Table 1. Growth performance data is presented in Table 3. There was no effect of dietary treatment on the final BW of pigs. The ADG, ADFI, and G:F for pigs fed basal diet with antibiotics or DFM did not differ from those of pigs fed the unsupplemented basal diet during the entire study period (days 0–21). However, the supplementation of the basal diet with antibiotics or DFM increased (*P* < 0.05) the G:F of pigs during the postchallenge period (from day 3 to 21) but did not affect the ADG and ADFI of pigs during this postchallenge period. The G:F for DFM*-*supplemented diet did not differ from that for the antibiotic-supplemented diet. Diarrhea data are presented in Figs. 1 and 2. Supplementation of the basal diet with antibiotics or DFM tended to decrease (*P* = 0.071) the frequency of diarrhea (fecal score of 3–6) from day 0 to 21. The frequency of diarrhea was 22% for basal diet, 12% for basal diet with antibiotics, and 15% for basal diet with DFM. In addition, the survival rate of the pigs fed the basal diet was 75%, whereas 100% survival was observed for the pigs fed the antibiotic or DFM-supplemented diet. In the basal diet, two out of seven pigs died 1 and 2 d postchallenge, and results from necropsy confirmed that this was due to the *E. coli* challenge.

**Table 2.** Analyzed composition of the experimental diets (as-fed basis)

	Diet^*a*^		
Item	NC	PC	DFM
Dry matter, %	89.96	89.74	89.92
Crude protein, %	21.29	21.61	21.51
Amino acid, %			
Lys	1.54	1.58	1.59
Met	0.60	0.59	0.60
Met + Cys	0.94	0.93	0.95
Thr	1.00	0.97	0.99
Trp	0.35	0.36	0.35
Arg	1.34	1.37	1.35
Ile	0.89	0.90	0.89
Leu	1.68	1.70	1.70
Val	1.09	1.08	1.09
His	0.56	0.57	0.53
Phe	0.98	0.99	0.99
Spore count *B. subtilis*, CFU/g	0.00	0.00	1.3E+06
Background *Bacilli*, CFU/g	5.7E+03	1.7E+03	<1E+03

^*a*^NC = negative control diet, PC = NC supplemented with 0.25% of antibiotics, and DFM = NC supplemented with 0.05% DFM product (*B. subtilis;* DSM 32540).

**Table 3.** Effect of different dietary treatments on growth performance of *E. coli*-challenged weaned pigs

	Diet^*a*^				
Item	NC	PC	DFM	SEM	*P*-value
BW, kg					
Day 0	8.02	8.00	8.62	0.299	0.28
Day 3	8.48	8.23	8.48	0.141	0.38
Day 7	8.54	8.55	8.68	0.294	0.97
Day 14	10.46	10.55	10.44	0.627	0.67
Day 21	14.30	14.38	14.95	0.960	0.63
ADG, kg					
Days 0–21	0.283	0.288	0.310	0.050	0.91
Days 3–21	0.308	0.333	0.347	0.048	0.86
ADFI, kg					
Days 0–21	0.429	0.378	0.403	0.050	0.73
Days 3–21	0.483	0.421	0.448	0.053	0.72
G:F, kg/kg					
Days 0–21	0.653	0.740	0.766	0.037	0.14
Days 3–21	0.633^b^	0.778^a^	0.785^a^	0.033	0.01

^ab^Within a row, means without a common superscript differ (*P* < 0.05).

^*a*^NC = negative control diet, PC = NC supplemented with 0.25% of antibiotics, and DFM = NC supplemented with 0.05% DFM product (*B. subtilis;* DSM 32540).

Data on the effects of diets on small intestinal histomorphology is shown in Table 4. The VH and VH:CD ratio of jejunum were increased (*P* < 0.001) by antibiotics or DFM supplementation. However, the CD was not affected by supplementation with antibioitcs or DFM. The jejunal VH and VH:CD ratio for the DFM*-*supplemented diet were greater (*P* < 0.05) than those fed the antibiotic-supplemented diet. Ileal VH was increased (*P* < 0.05) by DFM supplementation. However, CD and VH:CD ratio of ileum were unaffected by any of the two supplementations. The effects of diets on visceral organ weights and gastrointestinal pH of pigs at 21 d of age are presented in Table 5. The weights of the liver, stomach, small intestine, cecum, and colon were not affected by antibiotics or DFM supplementation. Spleen weight was increased (*P* < 0.05) by antibiotics supplementation. Moreover, the addition of antibioitcs to the basal diet reduced (*P* < 0.05) the ileal digesta pH value. Cecal digesta pH was unaffected by antibiotics or DFM supplementation.

**Table 4.** Effect of different dietary treatments on gut histomorphology of *E. coli*-challenged weaned pigs

	Diet^*a*^				
Item	NC	PC	DFM	SEM	*P*-value
Jejunum					
VH, µm	305^c^	406^b^	435^a^	2.131	<0.001
CD, µm	177	177	182	2.862	0.470
VH:CD, µm/µm	1.72^c^	2.29^b^	2.40^a^	0.037	<0.001
Ileum					
VH, µm	298^b^	389^ab^	437^a^	42.78	0.095
CD, µm	170	164	171	16.18	0.95
VH:CD, µm/µm	1.98	2.64	2.53	0.261	0.18

^abc^Within a row, means without a common superscript differ (*P* < 0.05).

^*a*^NC = negative control diet, PC = NC supplemented with 0.25% of antibiotics, and DFM = NC supplemented with 0.05% DFM product (*B. subtilis;* DSM 32540).

**Table 5.** Effect of different dietary treatments on visceral organ weights and gastrointestinal digesta pH of *E. coli*-challenged weaned pigs

	Diet^*a*^				
Item^*a*^	NC	PC	DFM	SEM	*P*-value
Organ weight, g/kg of BW					
Spleen	1.76^b^	2.53^a^	2.03^ab^	0.231	0.09
Liver	26.9	28.7	26.3	1.125	0.33
Stomach	7.31	8.88	8.00	0.575	0.18
Small intestine	35.4	41.2	41.2	2.918	0.29
Cecum	2.08	2.39	2.08	0.181	0.40
Colon	14.1	15.7	14.4	1.098	0.54
Digesta pH					
Ileal	6.63	6.02	6.26	0.176	0.08
Cecum	5.58	5.65	5.51	0.086	0.38

^ab^Within a row, means without a common superscript differ (*P* < 0.05).

^*a*^NC = negative control diet, PC = NC supplemented with 0.25% of antibiotics, and DFM = NC supplemented with 0.05% DFM product (*B. subtilis;* DSM 32540).

The effects of dietary treatment on cecal digesta VFA concentration on day 21 of the experiment are presented in Table 6. The concentrations of acetic acid, propionic acid, butyric acid, isobutyric acid, and isovaleric acid were not affected by antibiotics or DFM supplementation. Supplementation of the NC diet with antibiotics or DFM lowered (*P* < 0.05) the valeric acid concentration in cecal digesta.

**Table 6.** Effect of different dietary treatments on VFA in cecal digesta of *E. coli*-challenged weaned pigs

	Diet^*a*^				
Item	NC	PC	DFM	SEM	*P*-value
VFA concentration, mM/g of dry matter					
Acetic acid	2.49	2.58	1.87	0.525	0.531
Propionic acid	1.59	1.33	0.94	0.302	0.266
Butyric acid	0.63	0.60	0.57	0.119	0.930
Branched-chain VFA					
Isobutyric acid	0.03	0.04	0.02	0.008	0.173
Valeric acid	0.23^a^	0.09^b^	0.10^b^	0.040	0.051
Isovaleric acid	0.03	0.03	0.02	0.007	0.288
Molar ratios of VFA, %					
Acetic acid	50.7	54.9	52.4	2.022	0.426
Propionic acid	31.0	27.5	27.0	2.470	0.450
Butyric acid	12.9	14.3	16.8	1.856	0.268
Branched-chain VFA					
Isobutyric acid	0.47	0.97	0.41	0.189	0.141
Valeric acid	4.32^a^	1.47^b^	2.85^ab^	0.613	0.037
Isovaleric acid	0.61	0.84	0.53	0.165	0.420

^ab^Within a row, means without a common superscript differ (*P* < 0.05).

^*a*^NC = negative control diet, PC = NC supplemented with 0.25% of antibiotics, and DFM = NC supplemented with 0.05% DFM product (*B. subtilis;* DSM 32540).

## DISCUSSION

In the current study, the supplementation of the basal diet with antibiotics or DFM resulted in improved feed efficiency of pigs during the postchallenge period, which could partly be explained by the reduced frequency of diarrhea and increased jejunal VH by the supplementation. Diarrhea is positively correlated with increased water and nutrient secretion in GIT or is negatively correlated with nutrient digestion and absorption or both (O’Loughlin et al., 1991; Fairbrother et al., 2005); all these led to reduced feed efficiency. Also, diarrhea due to ETEC is positively correlated with immune response, which, in turn, is associated with an increase in the proportion of dietary energy and nutrients that are utilized for maintenance (immune response) and a decrease in the proportion of dietary energy and nutrients that are utilized for growth (skeletal tissue deposition; Kiarie et al., 2011) and, hence, reduced feed efficiency. Small intestinal VH is positively correlated with the surface area for nutrient absorption and, hence, the efficiency of dietary nutrient utilization (Pluske et al., 1996a; Wu et al. 1996). In addition to reducing diarrhea and increasing the VH, the DFM can improve the G:F by enhancing nutrient digestibility through the production of enzymes that digest carbohydrates, such as nonstarch polysaccharides (Tang et al., 2019). The improvement in the G:F of pigs due to the inclusion of antibiotics in diets fed in the current study was expected because antibiotics have been added in diets for weaned pigs to improve gut health and feed efficiency (Heo et al., 2013). Dietary antibiotics may improve the feed efficiency partly by suppressing the growth of certain intestinal microorganisms that are pathogenic or compete with the host for nutrients (Dibner and Richards, 2005; Li, 2017). The improvement in the G:F of pigs due to supplemental DFM in the current study is in agreement with previous studies using diets supplemented with DFM that contained *B. subtilis* (Guo et al., 2006; Wang et al., 2011; Lee et al., 2014). However, the results of the current study are contrary to those reported from the study of Walsh et al. (2007), who did not observe an increase in the feed efficiency of weaned pigs due to the supplementation of DFM that contained *B. licheniformis* and *B. subtilis* to diets at 0.05%. Bontempo et al. (2006) suggested that the efficacy of DFM with regard to improving growth performance of weaned pigs may partly depend on the dosage and composition of DFM. The DFM product fed in the current study contained *B. subtilis* and was added in diets at 0.05%, whereas the DFM product fed in the study of Walsh et al. (2007) contained *B. licheniformis* and *B. subtilis*. Thus, the differences among the studies with regard to the effects of DFM on feed efficiency could be explained by differences in the composition of DFM products. The G:F for DFM-supplemented diet did not differ from that of antibiotics-supplemented diet during the postchallenge period, implying that the dietary DFM product in the current study can improve the feed efficiency of *E. coli*-infected weaned pigs to that of antibiotic-containing diet.

Infection of weaned pigs with pathogenic strains of *E. coli*, including K88+ strain, causes diarrhea as the toxins produced by pathogenic *E. coli* lead to increased secretion of fluids into the small intestine and reduced (re)absorption of fluids (O’Loughlin et al., 1991; Fairbrother et al., 2005). The postweaning diarrhea is more severe during the first 2 wk postweaning (Fairbrother et al., 2005). In the current study, the frequency of diarrhea by *E. coli*-challenged weaned pigs was reduced by dietary inclusion of antibiotics or DFM. In the small intestine, most cells in villous have absorptive function, whereas most cells in the crypt have secretory function, implying that an increase in VH:CD ratio results in an increase in net absorption of nutrients and fluids (De Jonge, 1975; Woyengo et al., 2011; Park et al., 2020). Thus, the increase in the jejunal VH:CD ratio observed in the current study due to antibiotics or DFM supplementation could partly explain the reduction in the frequency of *E. coli*-derived diarrhea. The results from the current study are in agreement with those from previous studies that reported a reduction in diarrhea (or frequency) in weaned pigs fed DFM (Bhandari et al., 2008; Yang et al., 2014; Pan et al., 2017).

Cell proliferation occurs in the crypt and, hence, a decrease in VH:CD ratio indicate a net decrease in mitotic activity in mucosa and, hence, the weight of the small intestine (King et al., 2008). In the current study, however, the weight of the small intestine relative to the live weight of weaned pigs was unaffected by antibiotic or DFM supplementation despite the fact that VH:CD ratio was increased by the antibiotic or DFM supplementation. Kiarie et al. (2011) also did not observe a change in the weight of the small intestine relative to the live weight of weaned pigs due to the supplementation of diets with antimicrobial agents or DFM. Thus, it appears that the small intestinal VH:CD ratio can change without a change in the total weight of small intestinal relative to live BW. The weight of the large intestine (cecum and colon) relative to that of live BW was also unaffected by DFM supplementation, which could be attributed to the lack of effect of DFM on VFA production. The VFA, especially butyric acid, stimulates cell proliferation in the large intestine (Sakata, 1987; Kien et al., 2007). Similarly, Awad et al. (2009) did not observe any change in the weight of the large intestine relative to the live weight of weaned pigs due to the supplementation of diets with DFM.

One of the functions of the spleen in animals is to induce immunity in response to infection (Lewis et al., 2019). Thus, the size of the spleen in animals can potentially be increased due to infection, implying that the size of the spleen of *E. coli*-challenged pigs is expected to reduce due to the supplementation of diets with feed additives that alleviate the *E. coli* infection (Kiarie et al., 2011). In the current study, the supplementation of the NC diet with antibiotics increased the size of the spleen relative to the live BW, and the reason for this is not clear. The size of the spleen relative to live weight was not affected by the supplementation of the NC diet with DFM. It should be noted that DFM can alleviate GIT infections partly by stimulating the immune response (Grant et al., 2018), which may explain the lack of effect of DFM on the spleen. White blood cell production was not measured in the current study.

The VH in the small intestine of weaned pigs is positively correlated with small intestinal luminal energy and nutrients availability (Pluske et al., 1996b) and negatively correlated with small intestinal proliferation of pathogenic microorganisms (O’Loughlin et al., 1991). Thus, in the current study, the increase in jejunal VH:CD by DFM supplementation could have been due to an increase in the luminal availability of energy and other nutrients or reduced proliferation of the pathogenic microorganisms, such as ETEC, or both by the supplementation. The DFM supplementation could have increased the luminal availability of energy and other nutrients by increasing their digestibility (not measured in the current study) because feed intake was not affected by the DFM supplementation. As previously mentioned, the *B. subtilis* in the DFM product fed in the current study produces various fiber-degrading enzymes, including xylanase and cellulase. These fiber-degrading enzymes can hydrolyze the fiber in the upper part of the small intestine, thereby releasing fiber-encapsulated nutrients for digestion and absorption (Woyengo and Nyachoti, 2011).

The jejunal VH:CD for pigs fed the DFM-supplemented diet was greater than that of pigs fed the antibiotic-supplemented diet, implying that the DFM product fed in the current study was more effective than the antibiotics with regard to improving jejunal histomorphology of ETEC-challenged weaned pigs. The ileal VH:CD was unaffected by the antibiotic or DFM supplementation. Weaning stress and availability of nutrients in the lumen of the small intestine have a significant effect on the integrity of the upper part, but not the lower part, of the small intestine of weaned pigs (Wijtten et al., 2011), which could partly explain the limited effect of DFM on ileal VH:CD. Also, it could be possible that the upper part of the small intestine of weaned pigs is more colonized by food-borne pathogenic *E. coli* compared to the lower part, leading to a limited effect of the *E. coli* infection on the integrity of the lower part of the small intestine.

The DFM supplementation did not affect the acetic acid, propionic acid, and butyric acid production and, hence, the pH in the cecal digesta. It had been assumed that the DFM would increase VFA and, hence, reduce the pH in GIT because the *B. subtilis* in DFM product fed in the current study can promote fiber fermentation because it has xylanase activity (International Patent Application WO 2019/002471), and xylanase targets arabinoxylans that are the major nonstarch polysaccharides in corn (Knudsen, 2014). Diets fed in the current study were based on corn. However, the DFM may have increased the nutrients digestibility in the upper part of the small intestine (as evidenced by increased VH:CD without increased feed intake), leading to reduced availability of the substrate for fermentation in the lower part of the small intestine and in the large intestine. Results from previous studies have shown reduced hindgut digestibility or fermentation as a result of an increase in the digestibility of nutrients in the small intestine by fiber-degrading enzyme supplementation (Woyengo et al., 2016; Lee et al., 2018). Indeed, the DFM supplementation numerically reduced the cecal digesta concentration of the aforementioned VFA (acetic acid, propionic acid, and butyric acid) and significantly reduced the cecal digesta valeric acid concentration, which could support this aforementioned hypothesis. Valeric acid is a byproduct of protein fermentation (van Straalen and Tas, 2010). Thus, its reduced concentration in cecal digesta implies that the DFM increased protein digestion in the small intestine or inhibited the growth of protein-fermenting microorganisms in the cecum. The fermentation of proteins in the hindgut is negatively associated with gut health because the end products of protein fermentation, such as ammonia, indoles, and skatoles are toxic to animals (Heo et al., 2013). Thus, the DFM product fed in the current study may improve the health of pigs partly by reducing protein fermentation in the hindgut.

In conclusion, the supplementation of the basal diet with the DFM improved the feed efficiency, increased the jejunal VH and VH:CD, and decreased the frequency of diarrhea of weaned pigs that were challenged with K88 strain of *E. coli*. The feed efficiency of pigs fed the diet supplemented with DFM (*B. subtilis* DSM 32540 strain) did not differ from that of pigs fed the antibiotic-supplemented diet, whereas the jejunal VH and VH:CD for pigs fed the DFM-supplemented diet was greater than that of pigs fed the antibiotic-supplemented diet. Thus, the use of *B. subtilis* DSM 32540 strain containing DFM product in diets for weaned pigs under *E. coli* challenge may replace the use of antibiotics in diets to improve growth performance and gut histomorphology.
